# Drug-Disease Interactions Causing Hemorrhagic Cardiac Tamponade

**DOI:** 10.7759/cureus.117237

**Published:** 2026-09-30

**Authors:** Brian Torres-Mercado, Xavier Delgado-Lopez, Joseph Maldonado-Suarez, David R Bermudez Nunez, Juan F Rodriguez Acosta

**Affiliations:** 1 Cardiology, Mayagüez Medical Center, Mayagüez, PRI

**Keywords:** amiodarone, apixaban, cardiac tamponade, direct oral anticoagulants, drug–drug interaction, hemopericardium, hypothyroidism, myxedema, pericardial effusion, wolff–chaikoff effect

## Abstract

Hemorrhagic tamponade in severe hypothyroidism is an uncommon presentation that warrants evaluation for superimposed pathology. We present the case of a 57-year-old man with nonischemic cardiomyopathy, chronic amiodarone therapy, and atrial fibrillation on apixaban who developed advanced myxedema, complicated by a large hemorrhagic pericardial effusion and cardiac tamponade. This case highlights a rare but life-threatening convergence of amiodarone-induced severe hypothyroidism and potentiated anticoagulation, culminating in hemorrhagic tamponade. Recognition of drug-disease interactions is critical in anticoagulated patients with endocrine dysfunction who present with rapidly recurrent pericardial effusion.

## Introduction

Severe hypothyroidism is a well-established cause of pericardial effusion, occurring in up to 3-37% of patients [1]. These effusions typically accumulate gradually, are serous in nature, and rarely progress to cardiac tamponade because the pericardium adapts to slow distention [1]. Hemorrhagic conversion, however, is atypical and should prompt evaluation for alternative or superimposed etiologies, including malignancy, infection, autoimmune disease, myocardial rupture, or anticoagulation-related bleeding [2].

Amiodarone is a highly effective antiarrhythmic agent but is strongly associated with thyroid dysfunction due to its high iodine content and effects on thyroid hormone metabolism [3]. Chronic therapy may induce overt hypothyroidism, particularly in susceptible individuals. Concurrently, direct oral anticoagulants such as apixaban are increasingly prescribed in patients with cardiomyopathy and atrial arrhythmias. Apixaban metabolism depends in part on CYP3A4 and P-glycoprotein pathways, both of which may be inhibited by amiodarone, potentially increasing systemic anticoagulant exposure [4].

We present a case of rapidly recurrent hemorrhagic cardiac tamponade in the setting of amiodarone-induced myxedema and concomitant apixaban therapy, illustrating a multidimensional drug-disease interaction with life-threatening consequences.

## Case presentation

A 57‑year‑old man presented with four weeks of progressively worsening fatigue. His medical history included essential hypertension, prior immune thrombocytopenic purpura, remote cocaine use, and nonischemic cardiomyopathy with a left ventricular ejection fraction of 30%, for which an automatic implantable cardioverter‑defibrillator (AICD) had been placed three years earlier. His medications consisted of carvedilol, losartan, and amiodarone 250 mg twice daily, which was initiated several months prior to admission. The patient was also started on apixaban for atrial fibrillation several weeks prior to admission. On examination, his blood pressure was 110/70 mmHg with a regular heart rate of 103 beats per minute, and he exhibited pulsus paradoxus, dry skin with madarosis, and bilateral nonpitting edema. Cardiac auscultation revealed normal S1 and S2 with an S3 gallop, but no pericardial rub, and his lungs were clear. The differential diagnosis included hypothyroid‑related pericardial effusion, anticoagulation‑associated hemopericardium, malignancy, infectious pericarditis, and device‑related perforation. Transthoracic echocardiography demonstrated a large circumferential pericardial effusion with right atrial systolic and right ventricular diastolic collapse, findings consistent with cardiac tamponade physiology (Figures 1-2).

**Figure 1:**
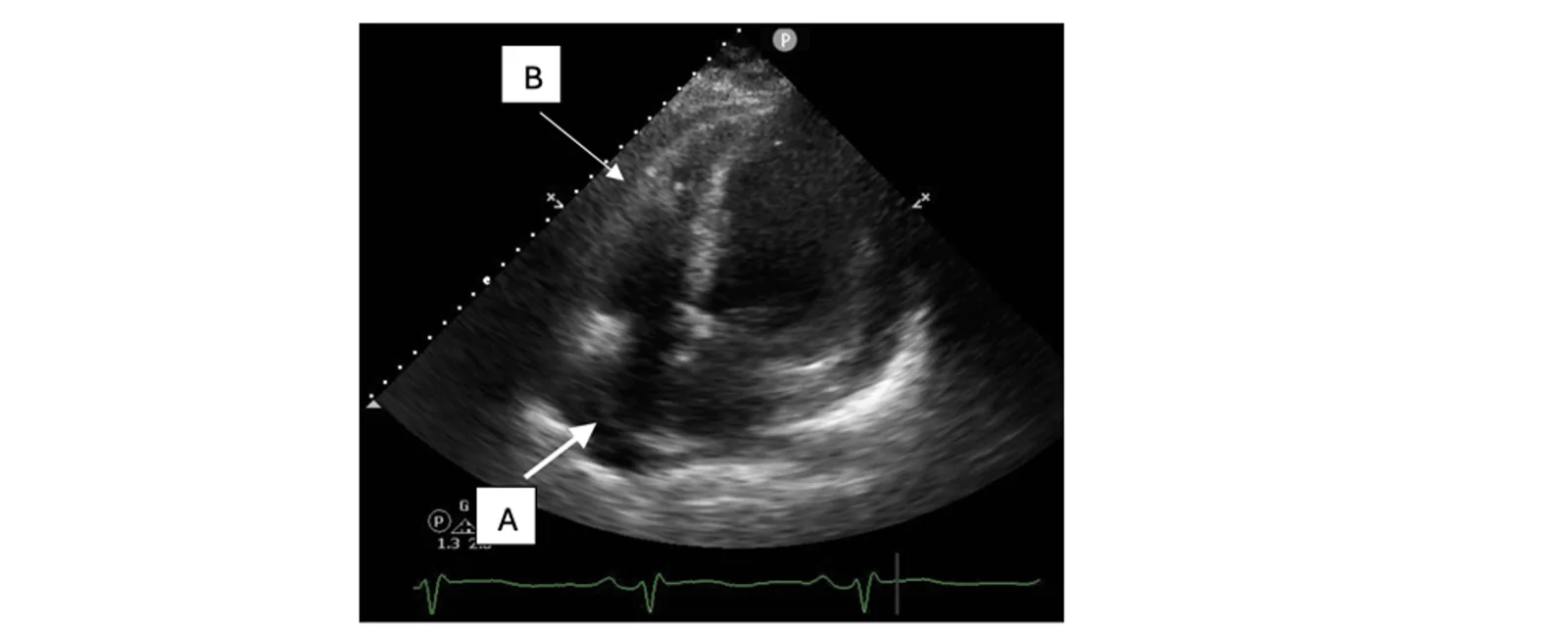
Transthoracic echocardiography demonstrating a large circumferential pericardial effusion with right atrial systolic collapse (A) and right ventricular diastolic collapse (B) consistent with cardiac tamponade.

**Figure 2:**
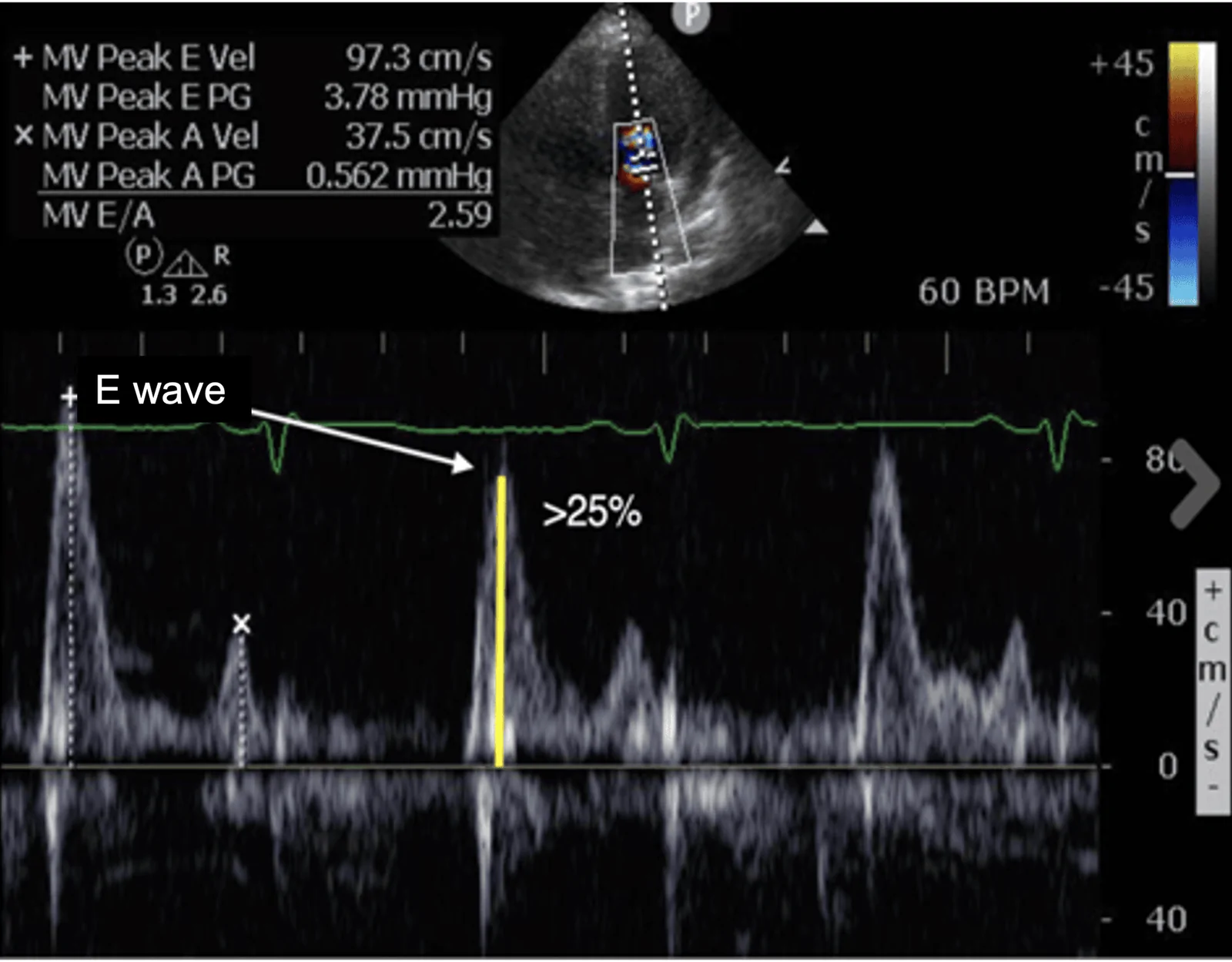
Mitral inflow E-wave velocity decreasing >25% with inspiration, characteristic of cardiac tamponade physiology.

Management and clinical course

Emergent pericardiocentesis drained 700 mL of hemorrhagic fluid, resulting in transient hemodynamic improvement. Within two hours, rapid reaccumulation due to drain obstruction required evacuation of an additional 500 mL. Pericardial fluid analysis revealed a hemorrhagic effusion with a protein level of 5.2 g/dL. Cultures grew *Pseudomonas aeruginosa*, *Proteus mirabilis*, and *Enterobacter cloacae* complex. Cytology was negative for malignancy. Laboratory results are summarized in Table 1, which showed leukocytosis and a normal platelet count. Autoimmune and viral studies were negative, although Coxsackie A IgG titers were elevated, suggestive of recent exposure. Toxicology screening, tuberculosis testing, and blood cultures were all negative. Thyroid function tests indicated severe primary hypothyroidism. The clinical and laboratory findings were consistent with advanced myxedema.

**Table 1:** Laboratory Findings AST: aspartate aminotransferase; ALT: alanine aminotransferase; IgG: immunoglobulin G; ​​​​​​​PT: prothrombin time; PTT: partial thromboplastin time; TSH: thyroid stimulating hormone

Laboratory	Results	Reference range
White blood cell count	20,000/µL	3.98-10.04 × 10³/μL
Platelet count	210,000/µL	163-369 x 10^3^/µL
Creatinine	1.63 mg/dL	0.70-1.20 mg/dL
AST	35 U/L	10-50 U/L
ALT	30 U/L	10-50 U/L
Alkaline phosphatase	43 U/L	40-129 U/L
Total bilirubin	1.86 mg/dL	0.0-1.2 mg/dL
Direct bilirubin	0.65 mg/dL	0.00-0.30 mg/dL
Indirect bilirubin	1.2 mg/dL	0.00-0.70 mg/dL
PT	16.1 sec	11.6-14.7 sec
PTT	33.2 sec	22.7-37.7 sec
Coxsackie A IgG titers	1:1,600	< 1:8
Toxicology screening	Negative	Negative
Tuberculosis testing	Negative	Negative
Blood cultures	Negative	Negative
TSH	152 µIU/mL	0.45 to 4.12 mIU/L
Free T4	0.225 ng/dL	0.93 – 1.7 ng/dL
Total T4	1.43 µg/dL	4.5-9.8 µg/dL
T3	41.4 ng/dL	80-220 ng/dL

Apixaban was discontinued on the day of admission after draining the hemorrhagic pericardial effusion, and amiodarone was discontinued once the results of thyroid function tests were available on the day after admission. Intravenous levothyroxine and broad-spectrum antibiotic therapy were initiated, followed by oral thyroid hormone replacement. A 42-day course of intravenous antibiotics was administered due to concern for contamination versus true infection, following infectious diseases service recommendations. The patient remained hemodynamically stable without further reaccumulation. Follow-up echocardiography demonstrated a small residual effusion (Figure 3). He was discharged with close cardiology and endocrinology follow-up. At discharge, TSH had decreased to 50 µIU/mL, with improvement in free T4 levels. The patient remained in normal sinus rhythm without recurrent atrial arrhythmia. In view of the life-threatening hemopericardium, the substantial bleeding risk, and the absence of an immediate rhythm-based indication, anticoagulation treatment was not resumed. The patient was subsequently referred for evaluation of left atrial appendage occlusion with a Watchman device (Boston Scientific Corporation, Marlborough, MA) as a potential strategy for long-term stroke-risk reduction while minimizing future exposure to systemic anticoagulation.

**Figure 3:**
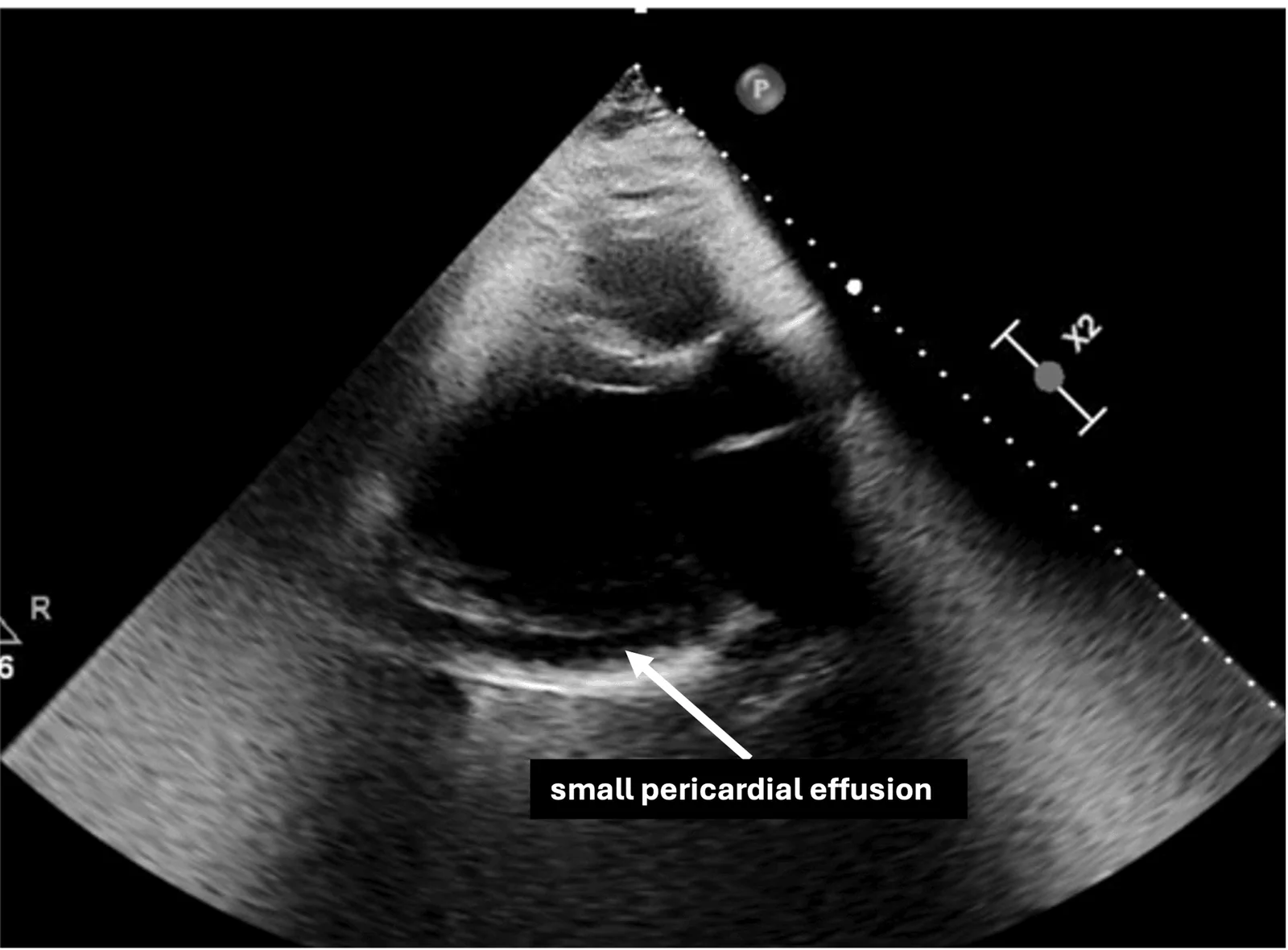
Small residual pericardial effusion after pericardiocentesis

## Discussion

This case demonstrates a rare but clinically important convergence of endocrine dysfunction, pharmacologic interaction, and hemorrhagic pericardial disease culminating in rapidly progressive cardiac tamponade. We propose a drug-disease interaction in which chronic amiodarone therapy-induced severe hypothyroidism with pericardial effusion formation, and concomitant apixaban therapy--potentially potentiated by metabolic inhibition--precipitated hemorrhagic conversion and hemodynamic compromise.

Amiodarone contains a high iodine content and is a well-recognized cause of thyroid dysfunction [3,5]. Through persistent activation of the Wolff-Chaikoff effect, excess iodine may inhibit thyroid hormone synthesis, resulting in overt hypothyroidism [3,5]. The markedly elevated TSH (152 µIU/mL) and profoundly reduced free T4 in this patient confirm severe hypothyroidism consistent with myxedema. Cardiovascular manifestations of advanced hypothyroidism include reduced myocardial contractility, increased systemic vascular resistance, and pericardial effusion [1].

Pericardial effusion is a recognized manifestation of severe hypothyroidism. The underlying mechanism involves increased capillary permeability, impaired lymphatic drainage, and interstitial accumulation of glycosaminoglycans, leading to gradual pericardial fluid accumulation [1]. Importantly, hemorrhagic effusion is not characteristic of isolated hypothyroidism and should prompt evaluation for additional causes of pericardial disease [1,2].

In this patient, severe untreated hypothyroidism likely resulted in chronic pericardial fluid accumulation, creating a permissive pericardial space. The defining feature of this case, however, was the hemorrhagic nature of the effusion and its rapid reaccumulation within two hours of initial drainage. Such rapid recurrence is concerning for ongoing intrapericardial bleeding rather than passive redistribution of fluid typical of a slowly accumulating hypothyroid-associated effusion [1,2].

The second critical component is the pharmacokinetic interaction between amiodarone and apixaban. Apixaban is metabolized in part by CYP3A4 and is a substrate for P-glycoprotein; inhibition of these pathways can affect apixaban plasma concentration [4]. Amiodarone is a moderate inhibitor of both pathways and therefore, concomitant administration may increase apixaban plasma concentrations, particularly in patients with advanced cardiomyopathy, possible hepatic congestion, and polypharmacy [4,6]. Although the patient was receiving guideline-directed standard dosing (5 mg twice daily), chronic amiodarone therapy may have reduced apixaban clearance, effectively increasing anticoagulant exposure.

Direct oral anticoagulants generally do not require routine laboratory monitoring, and clinically significant anticoagulant exposure may therefore remain unrecognized until bleeding occurs [4]. Apixaban intoxication often results in significantly elevated anti-factor Xa (anti-Xa) activity levels, but these were not measured in this case.

Spontaneous hemopericardium associated with direct oral anticoagulants has been described, although it remains uncommon [2]. In this context, we propose that anticoagulation facilitated spontaneous microvascular bleeding into a pre-existing hypothyroid-related effusion. The pathophysiologic sequence may therefore be conceptualized as shown in Figure 4.

**Figure 4:**
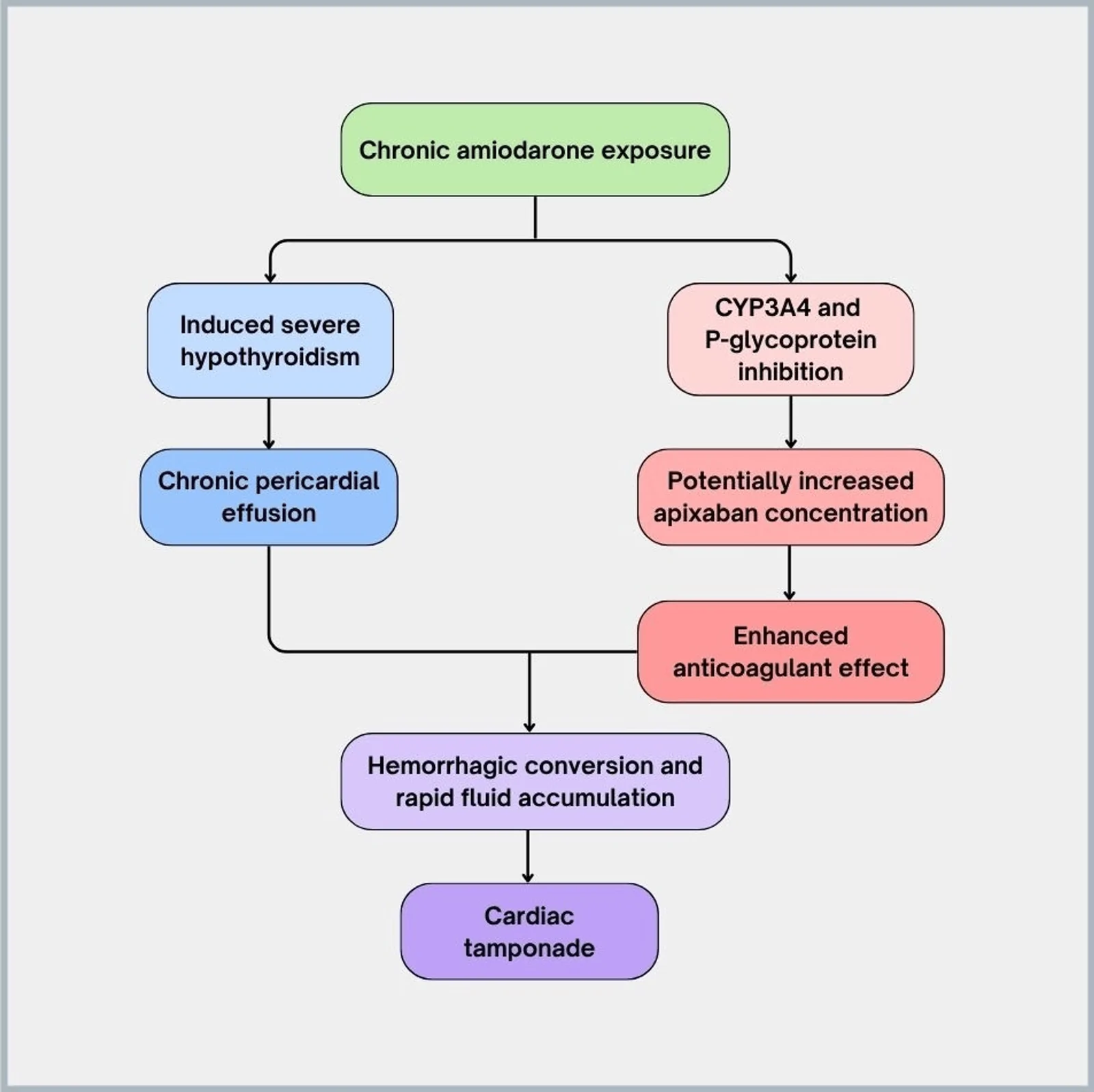
Proposed pathophysiologic sequence leading to cardiac tamponade in our case Original image created by Dr. Jomar Machuca.

This integrative mechanism explains the hemorrhagic fluid profile, the rapid reaccumulation following pericardiocentesis, and the absence of alternative etiologies [1,2,4,6].

Alternative causes of hemopericardium-including malignancy, infection, autoimmune disease, and structural cardiac injury-should be considered when evaluating a hemorrhagic pericardial effusion [2]. In this patient, despite having a history of immune thrombocytopenic purpura, platelet count was normal. In addition, qualitative platelet dysfunction or subclinical immune factors were considered but deemed less likely than the primary drug-disease interaction. Moreover, autoimmune testing was negative, viral studies did not demonstrate an active process, and echocardiography did not demonstrate structural disruption. The combination of profound biochemical hypothyroidism and exposure to potentially interacting pharmacotherapies therefore provides the most plausible explanation for the observed clinical course.

Clinically, this case underscores several important considerations. First, thyroid function should be monitored in patients receiving chronic amiodarone therapy because clinically significant thyroid dysfunction may develop during treatment [3,5]. Second, clinicians should remain vigilant regarding drug-drug interactions involving direct oral anticoagulants. Although amiodarone is not formally contraindicated with apixaban, concomitant therapy may affect anticoagulant exposure and should be considered when assessing individual bleeding risk [4,6]. Third, rapid reaccumulation of pericardial fluid following drainage should prompt consideration of active hemorrhage, particularly in anticoagulated individuals [2]. Finally, large pericardial effusions in anticoagulated patients with endocrine disorders should not be attributed to a single etiology without careful evaluation for superimposed hemorrhagic complications [1,2].

This case highlights how a typically indolent hypothyroid pericardial effusion can evolve into a life-threatening emergency when compounded by pharmacologic interaction. Recognition of this multidimensional mechanism is essential in patients with cardiomyopathy and polypharmacy to prevent catastrophic hemodynamic deterioration.

In addition to the proposed drug-disease interaction, three additional clinical findings further contextualize the complexity of this case.

First, the markedly elevated Coxsackie A IgG titer (1:1600) suggests recent viral exposure. Although IgG seropositivity alone does not establish active infection, high titers may reflect a recent or resolving viral process. Enteroviral infections, including Coxsackie species, are well-recognized causes of myopericardial inflammation. It is therefore plausible that a recent viral insult contributed to baseline pericardial inflammation or capillary fragility, thereby lowering the threshold for hemorrhagic conversion in the setting of anticoagulation. However, the absence of systemic inflammatory features, lack of myocardial involvement on imaging, and the clinical predominance of severe biochemical hypothyroidism argue against viral pericarditis as the primary driver. Instead, recent viral exposure may have acted as a permissive inflammatory substrate rather than the principal etiology.

Second, pericardial fluid cultures grew *Pseudomonas aeruginosa*, *Proteus mirabilis*, and *Enterobacter cloacae*. The polymicrobial Gram-negative profile is atypical for primary bacterial pericarditis, which more commonly presents with purulent effusion, systemic toxicity, and hemodynamic instability disproportionate to tamponade physiology. In this case, the drained fluid was hemorrhagic rather than purulent, and the patient lacked septic features. The growth of multiple enteric organisms raises concern for procedural contamination introduced during emergent pericardiocentesis, particularly given the bloody, non-purulent nature of the fluid and the urgent circumstances under which drainage was performed. While secondary inoculation cannot be definitively proven, the microbiologic pattern and clinical context make primary bacterial pericarditis unlikely. Importantly, true bacterial hemopericardium would be expected to demonstrate persistent inflammatory response and progressive septic physiology, neither of which were observed.

Third, the presence of an AICD required consideration of device-related complications, including lead perforation, delayed erosion, or device-associated endocarditis with secondary pericardial involvement. However, there was no history of recent device implantation or manipulation, no echocardiographic evidence of lead perforation, and no imaging or microbiologic findings suggestive of infective endocarditis. The absence of vegetations, pocket infection, or bacteremia further reduces the likelihood of device-mediated hemopericardium. Thus, although an AICD represents a potential structural risk factor in cases of hemorrhagic pericardial effusion, available evidence does not support it as the inciting mechanism in this patient.

When integrated into the broader pathophysiologic framework, these additional factors do not supersede the primary drug-disease interaction; rather, they refine the differential diagnosis.

Collectively, this case illustrates how overlapping endocrine dysfunction, anticoagulant pharmacokinetics, potential inflammatory priming, and invasive intervention can intersect in a critically ill patient. Careful mechanistic synthesis--rather than attribution to a single abnormal finding--is essential when evaluating rapidly recurrent hemorrhagic pericardial effusion [1,2,4,6].

## Conclusions

This case illustrates a rare but clinically significant combination of severe endocrine dysfunction, drug interaction, and hemorrhagic pericardial disease, leading to rapidly progressive cardiac tamponade. The case demonstrates that large pericardial effusions are often multifactorial, particularly in patients with cardiomyopathy and polypharmacy. It further underscores the importance of routine thyroid function testing during long-term amiodarone therapy and careful monitoring for drug interactions with direct oral anticoagulants, as these may elevate bleeding risk even with guideline-directed dosing. Rapid reaccumulation of pericardial fluid after drainage should prompt suspicion for ongoing hemorrhage in anticoagulated individuals. Early detection of drug-disease interactions and timely correction of reversible factors may prevent hemodynamic deterioration and improve clinical outcomes.
